# Prebiotics enhance the immunomodulatory effect of *Limosilactobacillus fermentum* DALI02 by regulating intestinal homeostasis

**DOI:** 10.1002/fsn3.4361

**Published:** 2024-07-28

**Authors:** Longfei Zhang, Xiaoxiao Liu, Yang Liu, Xinyi Cheng, Mingze Xu, Hengxian Qu, Wenqiong Wang, Ruixia Gu, Dawei Chen

**Affiliations:** ^1^ College of Food Science and Technology Yangzhou University Yangzhou China; ^2^ Key Laboratory of Dairy Biotechnology and Safety Control Yangzhou China

**Keywords:** gut microbiota, immunomodulatory, prebiotics, probiotics

## Abstract

The colonization ability of *Limosilactobacillus fermentum* DALI02 and the promoting effect of fermented prebiotics have been studied. This study aims to evaluate the systemic immunomodulatory effects of DALI02 and DALI02 + Prebiotics on cyclophosphamide‐induced immunosuppressed rats. We found that DALI02 and DALI02 + Prebiotics, especially DALI02 + Prebiotics, exhibited significant restorative effects on the immunocompromised state in rats (*p* < .05). Specifically, both of them promoted the recovery of body weight and immune organ function, enhanced the proliferative capacity of immune cells, and effectively reduced the levels of interleukin 6 (IL‐6) and tumor necrosis factor‐α (TNF‐α). Furthermore, both of them significantly reduced the levels of lipopolysaccharide (LPS) and D‐lactic acid in the blood (*p* < .05). Principal coordinate analysis (PCoA), principal component analysis (PCA), and unsupervised cluster analysis revealed that DALI02 and DALI02 + Prebiotics group were more similar to the blank group at the genus level of the gut microbiota. At the level of short‐chain fatty acids (SCFAs), DALI02 + Prebiotics and blank group belonged to Cluster 3. These results suggested that the intervention with DALI02 and DALI02 + Prebiotics effectively modulated the structure of the gut microbiota, and DALI02 + Prebiotics restored the dysregulation of SCFAs. In summary, DALI02 and DALI02 + Prebiotics possess immunomodulatory functions, with the latter showing superior effects.

## Graphical abstract

The systemic immunomodulatory effects of *Limosilactobacillus fermentum* DALI02 on cyclophosphamide‐induced immunosuppressed rats have been shown. DALI02 fermentation compound prebiotics can improve the immunomodulatory effect of the strain, through more effective regulation of intestinal flora and intestinal metabolites.

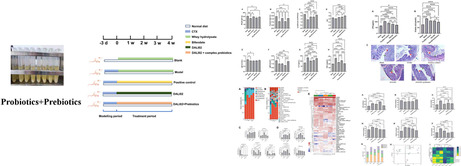

## 1 INTRODUCTION

Probiotics are living microbes that produce health benefits to the host when ingested in sufficient doses (Hill et al., 2014). They possess health benefits, such as repairing oxidative stress damage, activating the immune system, regulating systemic immune inflammation, assisting in lowering blood sugar and blood lipid levels, and alleviating liver damage associated with obesity (Vera‐Santander et al., 2023). Among them, immunomodulatory effect is one of the main research hotspots of probiotic characteristics of strains. Previous studies have shown that probiotics can effectively reduce the secretion of pro‐inflammatory factors (interleukin 6 (IL‐6) and tumor necrosis factor‐α (TNF‐α)) (Jorjao et al., 2015) and increase the expression of anti‐inflammatory factors (interleukin 10 (IL‐10) and interleukin 13 (IL‐13)) (Kim et al., 2019) in intestinal cells of immunocompromised rats. Probiotics have the capacity to modulate the gut microbiota composition, which can ameliorate the dysbiosis of the intestinal microbiome that arises from immunosuppression (Zeng et al., 2023). Previous studies have indicated that dietary supplementation with probiotics can effectively regulate the dysbiosis of the gut microbiota, increasing the abundance of *Lactobacillus* and *Bifidobacterium* at the genus level (Wang, Zeng, et al., 2024). Furthermore, the beneficial substances, specifically short‐chain fatty acids (SCFAs), secreted by probiotics in the gut play a significant role in intestinal function. Studies have shown that the intake of probiotics can increase the content of SCFAs in the intestine, including acetic acid, butyric acid, and propionic acid (Yue et al., 2020). Acetic acid, propionic acid, and butyric acid can activate the expression of tight junction proteins in colonic cells, thereby enhancing intestinal barrier function (Feng et al., 2018). This process effectively prevents harmful substances in the gut, such as lipopolysaccharides (LPS) and D‐lactic acid, from entering the bloodstream and thereby circumvents the occurrence of systemic inflammatory responses (Zhang et al., 2022). Therefore, dietary supplementation of probiotics to regulate the immune function of the body is one of the effective means to reduce inflammatory reaction.

Prebiotics are a class of substances that cannot be absorbed by the human gut but can be utilized by probiotics (You et al., 2022). Ingestion of prebiotics will provide fermentation substrates for beneficial bacteria in the gut and produce beneficial substances, especially SCFAs. Prebiotics can also promote the proliferation of beneficial bacteria in the intestinal tract, such as *Lactobacillus*, *Bifidobacterium*, and so on (Yu et al., 2023). In addition, it can specifically improve the growth activity, fermentation ability, and functional characteristics of the strain. Many studies have shown that probiotics have a certain preference for the use of prebiotics. Xu et al. found that amylopectin oligosaccharides and chitosan oligosaccharides were selectively utilized by *Lactobacillus* and *Bifidobacterium* strains, resulting in enhanced bacterial growth and lactic and acetic acid production (Xu et al., 2021). Currently, many studies have demonstrated the beneficial effects of single prebiotics in vitro and in vivo. However, there are few studies on the combination of prebiotics and the synergistic effects of prebiotics and probiotics.

Many studies have shown that the adhesion ability of probiotics is one of the important conditions for its health function. In the previous study, we characterized the probiotic potential of *Limosilactobacillus fermentum* DALI02, especially its high adhesion ability. The combination of prebiotics, which can improve the adhesion ability of DALI02, was optimized in vitro, and the effect of the compound prebiotics fermented by DALI02 on strain adhesion and colonization was verified in vivo (Liu et al., 2023). Currently, the immunomodulatory function of DALI02 in vivo and the effect of the fermentation prebiotics combination on the immunomodulatory effect of DALI02 have not been deeply studied.

Therefore, in this study, the immunocompromised rat model was established by injection of cyclophosphamide. The body weight, immune organ index, serum levels of inflammatory and immune factors, histopathological changes and intestinal environmental changes, including intestinal barrier function, intestinal flora structure, and intestinal SCFAs levels in different intervention groups, were measured to evaluate the immunomodulatory effect of DALI02 and the synergistic effect of compound prebiotics. This discovery is helpful to obtain probiotics with high adhesion ability and immunomodulatory potential, and to obtain prebiotic combinations with enhanced probiotic effect, so as to provide a theoretical basis for the development of symbiotic functional foods.

## 2 MATERIALS AND METHODS

### 2.1 Bacterial strains and cell strains

The *L. fermentum* DALI02 was isolated from traditionally fermented dairy products at the Key Laboratory of Dairy Biotechnology and Safety Control in Jiangsu Province. It is stored at the China General Microbiological Culture Collection Center (No. CGMCC 16064).

### 2.2 Preparation of whey hydrolysate and prebiotics

Whey hydrolysate and prebiotics were prepared via previous method (Liu et al., 2023). Ten percent whey protein powder (Fonterra, New Zealand) was adjusted to pH 7.00 with sodium hydroxide (NaOH) (Sinopharm, China, 0.1 mol/L). Then papain (Solarbio, Beijing, China) was added to skim milk and the enzyme activity reached 6000 U/g for hydrolysis. The hydrolysis temperature was 65°C, the hydrolysis time was 2 h, and the degree of hydrolysis was 20% under this condition. After that, the enzyme was killed at a high temperature (85°C, 30 min). The supernatant was obtained by centrifugation (6500 rpm (revolutions per minute) for 15 min). The supernatant was heat‐treated at 105°C for 10 min and stored at 4°C.

Fructose oligosaccharides, galactose oligosaccharides, inulin, stachyose, and xylooligosaccharides obtained from Jinfuyuan Biotechnology (Shenzhen, China) were added to the above enzymatic hydrolyzed whey according to the mass ratio of 0.05%, 0.20%, 0.30%, 0.20%, and 0.30% (w/w enzymatic hydrolyzed whey). The supernatant was heat‐treated at 105°C for 10 min. *L. fermentum* DALI02 was inoculated into the hydrolysate and the hydrolysate containing complex Prebiotics at the inoculation amount of 3% (v/w). The fermentation temperature was 37°C and the fermentation time was 24 h. The concentration of DALI02 in fermentation broth was adjusted to 1 × 10^9^ cfu/mL (colony‐forming units per milliliter), respectively.

### 2.3 Animal experiment design

The rats (4 weeks old, male, Wistar, 140–160 g) were provided by Yangzhou University Medical Animal Experiment Center (Yangzhou, China, Permission Number: SCXK 2016‐0006). The ambient temperature was 26 ± 1°C and the humidity was 55 ± 5% under a 12 h light–dark cycle with free access to water and food. Feed was supplied by Jiangsu Xietong Pharmaceutical Bio‐engineering Co., Ltd. (product number: XT93M).

**FIGURE 1:**
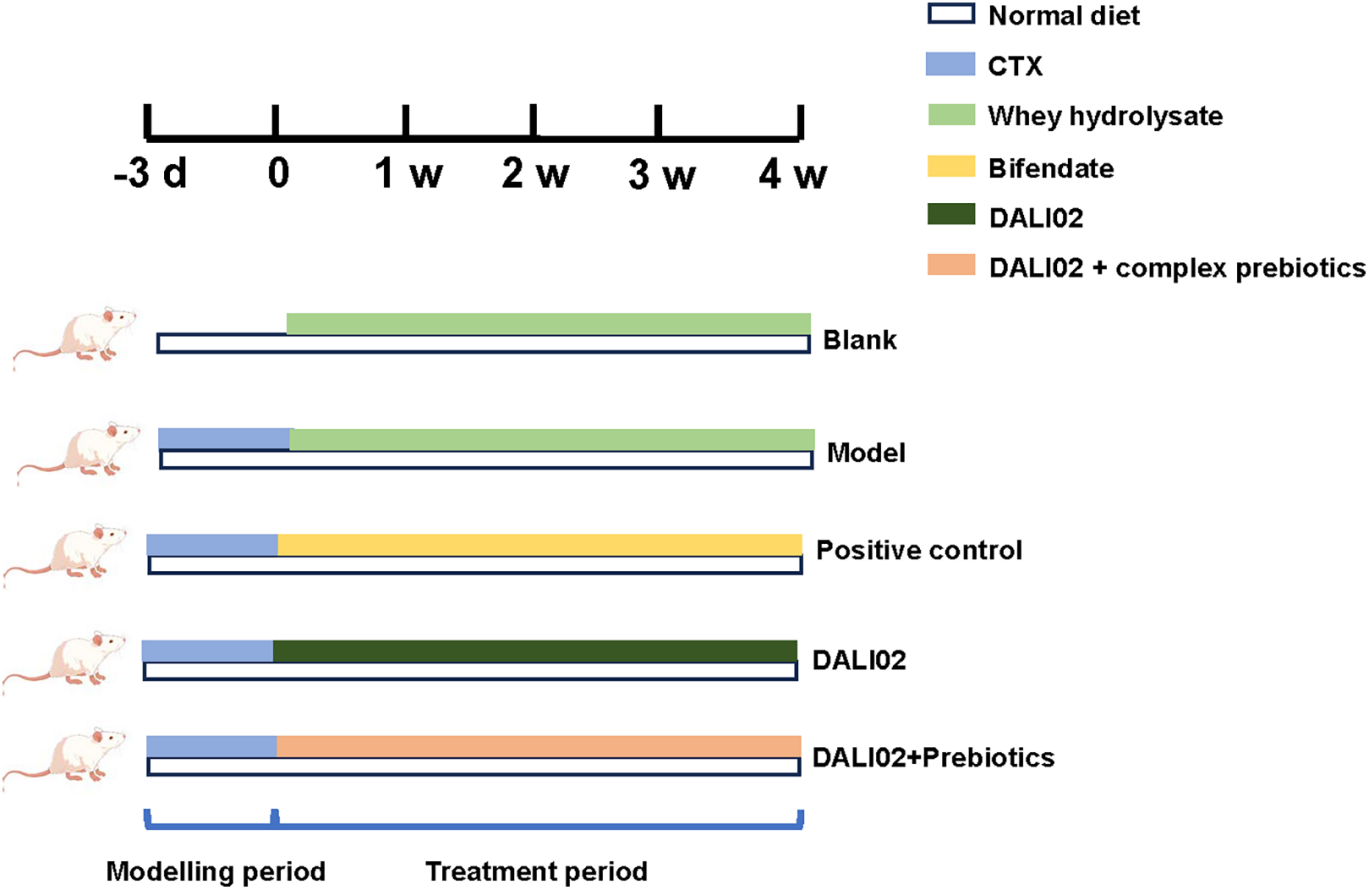
Diagrammatic sketch of the rats' experiment.

The rats were randomly divided into six groups after adaptation (*n* = 5). The details are shown in Table 1. Except the blank group, the other groups were injected with cyclophosphamide to establish the model of immunocompromised rats. The dosage of the cyclophosphamide was 10 mg/kg and the modeling period was 3 days. After the 3 days, we mainly detected the levels of IL‐6 and TNF‐α in rat blood to confirm the success of immunodeficiency model. The experimental design is shown in Figure 1.

**TABLE 1:** General situation of animal grouping and intragastric administration.

Group name	Intragastric sample	Feeding amount
Blank	Whey hydrolysate	1.8 mL/100 g d^−1^
Model	Whey hydrolysate	
Positive control	Bifendate pills	
DALI02	Fermented whey hydrolysate	
DALI02 + Prebiotics	Fermented whey hydrolysate with prebiotics	

After intragastric intervention, the rats were anesthetized, eyeballs were removed, and blood was collected. Serum samples were collected by centrifugation (3000 × g, 10 min, 4°C). Spleen and thymus were dissected and weighed. Tissue index (%) was calculated according to tissue mass (G)/body weight (G). The contents of small intestine, cecal tissue, and cecum were collected, and the contents of SCFAs were analyzed and stored at −80°C.

### 2.4 Pathological observation of colon in rats

The colon tissue was fixed with 4% paraformaldehyde, embedded in paraffin, and cut into 4‐μm thick sections. The fixed slices were dewaxed with xylene, hydrated with gradient ethanol, and soaked in distilled water. Then the slices were stained with hematoxylin–eosin (HE) staining solution (Beijing Solarbio Science & Technology Co. Ltd., Beijing, China). After staining, the slices were soaked in ethanol, n‐butanol, and xylene sequentially for dehydration. The morphological changes of the colon of rats in each group could be observed after staining of paraffin sections by HE.

### 2.5 Determination of spleen lymphocyte proliferation ability

Spleen lymphocyte proliferation ability was measured according to the method provided in the assay kit (Beijing Solarbio Science & Technology Co. Ltd., Beijing, China).

### 2.6 Determination of immunologically active substances

Immunoglobulin A (IgA), Immunoglobulin G (IgG), Immunoglobulin M (IgM), and Immunoglobulin A/Immunoglobulin G (A/G) were measured by a Model 7020 fully automated biochemical analyzer (Hitachi, Tokyo, Japan); interleukin 6 (IL‐6) and tumor necrosis factor‐α (TNF‐α) were measured according to the method provided in the assay kit (Shanghai Hualan Chemical Technology Co., Ltd., Shanghai, China).

### 2.7 Gut microbiota analysis

The colon contents of each group of rats after dissection were collected for DNA extraction. The gut sequencing analysis was completed by Shanghai Meiji Biotechnology Co. Ltd. (Shanghai, China). Total DNA was extracted from each sample utilizing the FastDNA® Spin Kit for Soil (MP Biomedicals, USA). The DNA quality and concentration were assessed using 1% agarose gel electrophoresis and a NanoDrop® ND‐2000 spectrophotometer (Thermo Scientific Inc., USA). The V3–V4 regions of bacterial 16S recombinant DNA (rDNA) were amplified and subsequently sequenced on an Illumina MiSeq platform (USA). The optimized sequences, obtained after quality control and merging, were denoised using the DADA2 plugin (or Deblur plugin) within the Qiime2 pipeline. The sequence data have been deposited in the National Center for Biotechnology Information (NCBI) Sequence Read Archive (SRA) database and were analyzed using the Majorbio Cloud Platform, a free online resource. The spliced high‐quality sequences were classified and based on the composition of the bacterial community, the composition and distribution of the intestinal flora of each group of rats were analyzed at the three classification levels of phylum, family, and genus.

### 2.8 Determination of short‐chain fatty acids (SCFAs) in feces

Short‐chain fatty acids were determined with a GC–MS 7890A/5975C ultra mass spectrometer (Agilent, USA) equipped with an Agilent DB‐WAX (Agilent, 30 m × 0.25 mm ID × 0.25 μm). The injection volume was 1 μL, with a split ratio of 10:1. The content of SCFAs was calculated based on standard calibration curves.

### 2.9 Statistical analysis

The results were recorded as mean values ± standard deviation. The significance of data in each group was compared with one‐way analysis of variance (ANOVA) in SPSS 17.0 software.

## 3 RESULTS

### 3.1 Dynamic changes in body weight and immunity in rats

**FIGURE 2:**
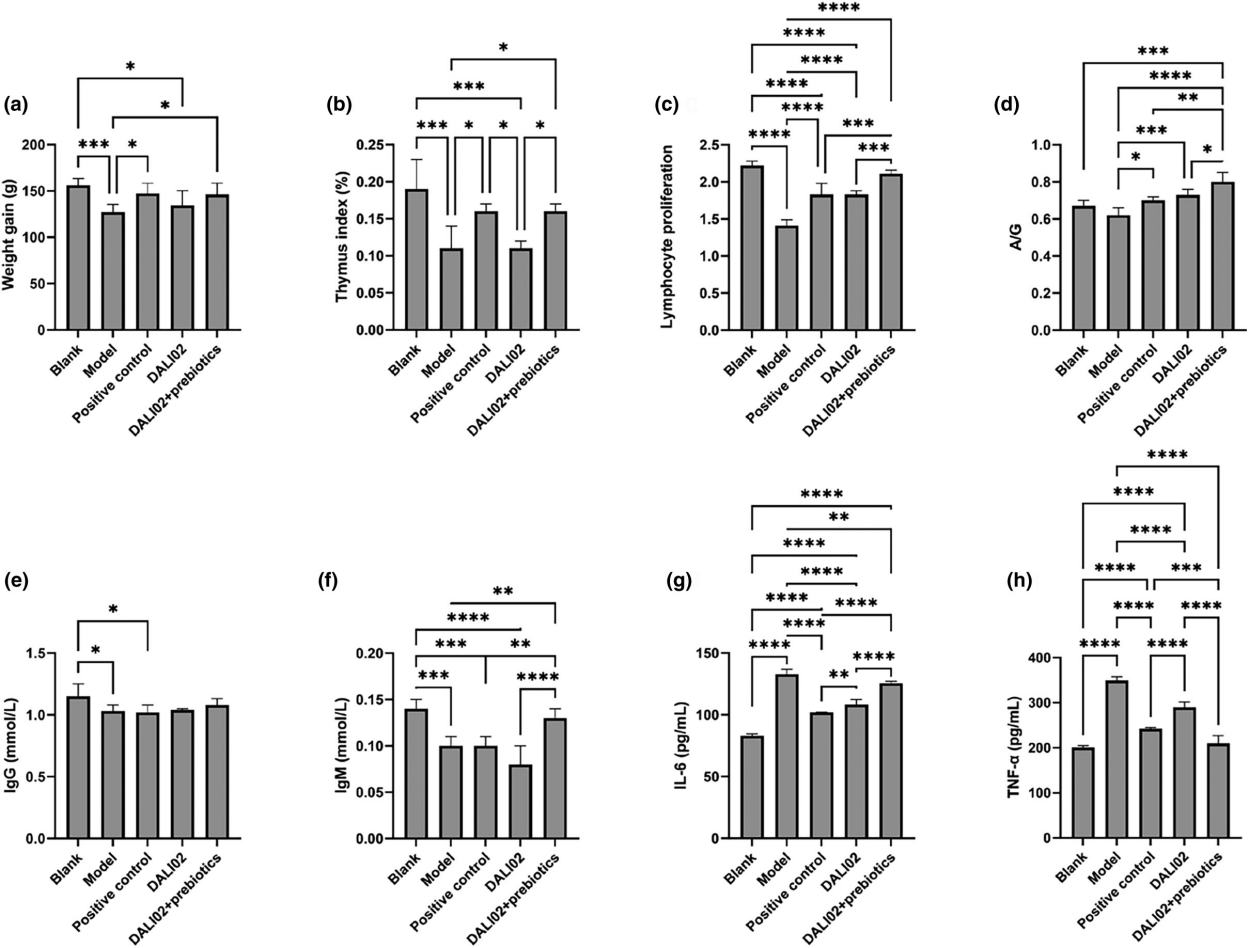
Comparison of body weight and immune activities. (a) Total weight gain. (b) Thymus index. (c) Splenic lymphocyte proliferation ability. (d–h) Serum levels of immunoglobulins. Ratio of serum albumin to globulin (A/G), IgG and IgM, immune factor levels of IL‐6 and TNF‐α. All data are means ± SEM, *n* = 5 per group. *, **, ***, **** Between different groups represents a significant difference (*p* < .05).

The changes in weight gain, immune organs, immunoglobulins, and immune factors in different intervention groups are shown in Figure 2. The body weight gain was 156.05 ± 7.52 g in the blank group and 127.33 ± 8.27 g in the model group (Figure 2a), which indicated that the injection of cyclophosphamide (CTX) could significantly inhibit the weight gain of rats (*p* < .05). However, the intervention of DALI02 and DALI02 + Prebiotics could alleviate this phenomenon. The weight gain was 134.50 ± 15.77 g in the DALI02 group and the weight gain was 146.25 ± 12.41 g in the DALI02 + Prebiotics group. The latter could significantly improve the weight change caused by CTX (*p* < .05). The thymus index and spleen lymphocyte proliferation showed the same trend (Figure 2b,c). The thymus index and spleen lymphocyte proliferation in the DALI02 + Prebiotics group were significantly higher than those in the model group and DALI02 group (*p* < .05), but not significantly different from those in the blank group (*p >* .05). The dynamic changes suggest that CTX has an impact on the immune organs and immune cells of rats. DALI02 + Prebiotics and DALI02 can restore the immune organ damage and restore body weight caused by CTX, and DALI02+ Prebiotics have better regulatory effects.

**FIGURE 3:**
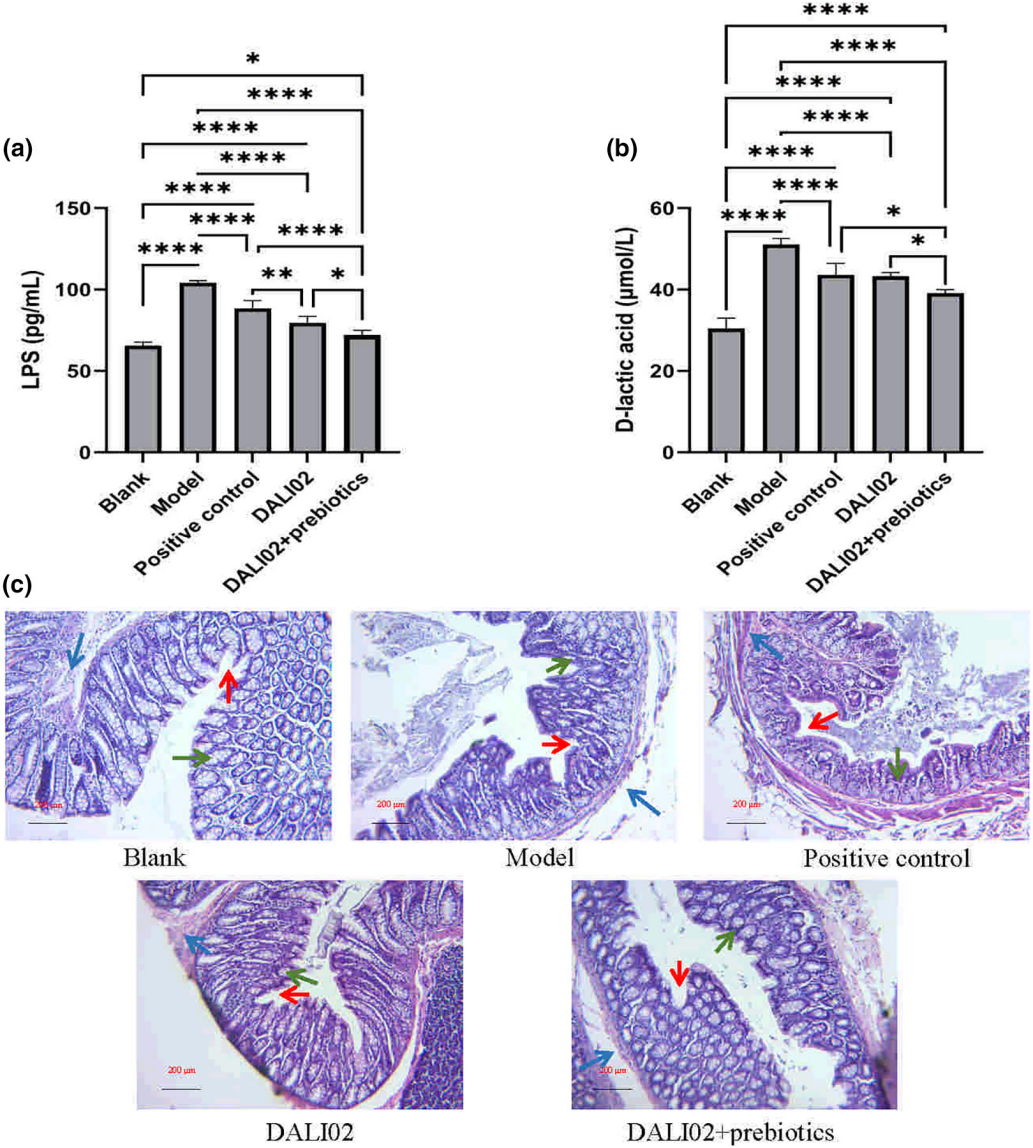
Changes in intestinal barrier function. (a) Changes in LPS content. (b) Changes in D‐lactic acid content. (c) Tissue sections of rat colon (×100). Red: Crypt Green: Goblet cells. Blue: Inflammatory cell infiltration. All data are means ± SEM, *n* = 5 per group. *, **, **** Between different groups represents a significant difference (*p* < .05).

As shown in Figure 2d–h, the levels of immunoglobulin, including A/G ratio, IgG and IgM in the model group, were significantly lower than those in the blank group (*p* < .05). This showed that CTX has a damaging effect on immunoglobulins in the immune system of rats. The A/G ratio in the model group was 0.62 ± 0.04, whereas the DALI02 group and the DALI02 + Prebiotics group exhibited A/G ratios of 0.73 ± 0.03 and 0.80 ± 0.05, respectively (Figure 2d). Both DALI02 and DALI02 + Prebiotics significantly modulated the A/G levels (*p* < .05). As shown in Figure 3f, the content of IgM in the DALI02 + Prebiotics group was significantly higher than that in the model group (*p* < .05), which was 0.13 ± 0.01 mmol/L, but there was no significant difference between the DALI02 group and the model group (*p >* .05). After intervention, the level of IgG increased, but there was no significant difference. These results indicated that DALI02 + Prebiotics modulated the levels of immunoglobulins more significantly and comprehensively than the DALI02 group alone.

As can be seen from Figure 2g,h, CTX can significantly increase the levels of pro‐inflammatory cytokines IL‐6 and TNF‐α in serum of rats (*p* < .05), reaching 132.85 ± 3.78 pg/mL (picograms per milliliter) and 349.31 ± 7.80 pg/mL, respectively. DALI02 and DALI02 + Prebiotics could significantly regulate the levels of IL‐6 and TNF‐α (*p* < .05). The content of IL‐6 decreased to 108.31 ± 4.06 pg/mL and 125.7 ± 1.30 pg/mL and the content of TNF‐α decreased to 289.06 ± 12.93 pg/mL and 209.43 ± 17.64 pg/mL, respectively. These results suggested that DALI02 + Prebiotics and DALI02 can significantly reduce the level of inflammatory factors and reduce the inflammatory response caused by CTX.

### 3.2 Effects on intestinal barrier function

After the 4‐week intervention, fresh blood was taken from rats in different groups, and the levels of LPS and D‐lactic acid in their serum were measured. The results are shown in Figure 2a,b. The contents of LPS and D‐lactic acid in serum of rats in the model group were 104.16 pg/mL and 51.09 μmol/L (micromoles per liter), respectively, which were significantly higher than those in the blank group (*p* < .05). The results showed that the intestinal permeability of rats in the model group increased, which made the metabolic harmful substances LPS and D‐lactic acid enter the blood, which may be caused by the intake of cyclophosphamide to damage the integrity of intestinal barrier in rats. After intervention with DALI02 and DALI02 + Prebiotics, the levels of LPS and D‐lactic acid in serum of rats were significantly lower than those in the model group (*p* < .05), especially in the DALI02 + Prebiotics group. The contents of LPS and D‐lactic acid were 72.17 pg/mL and 39.12 μmol/L, respectively. But there was still significant difference compared with the blank group (*p* < .05).

As can be seen from Figure 3c, the colonic tissue of rats in the blank group showed normal mucosa and intact epithelial tissue. The goblet cells were neatly arranged and the intestinal villi were long and unbroken. After injection of CTX, the area of intestinal cavity became larger, intestinal villi shortened and ruptured, crypt shrank and abscess, goblet cells cluttered and decreased, submucosal edema and inflammatory cell infiltration. After drug treatment, the morphology of crypt and goblet cells in rat colon was improved. Similarly, after the intervention of DALI02 and DALI02 + Prebiotics, the colon injury of rats was alleviated to a certain extent, such as the increase of crypt depth, the increase of goblet cells, and the decrease of inflammatory cell wetting. These figures showed that after the intervention, the intestinal injury caused by cyclophosphamide injection has been alleviated, and the recovery of intestinal mucosa and goblet cells provides a material basis for the exertion of intestinal barrier function. This is consistent with the results of LPS and D‐lactic acid.

### 3.3 Dynamic changes of richness and diversity in gut microbiota

**FIGURE 4:**
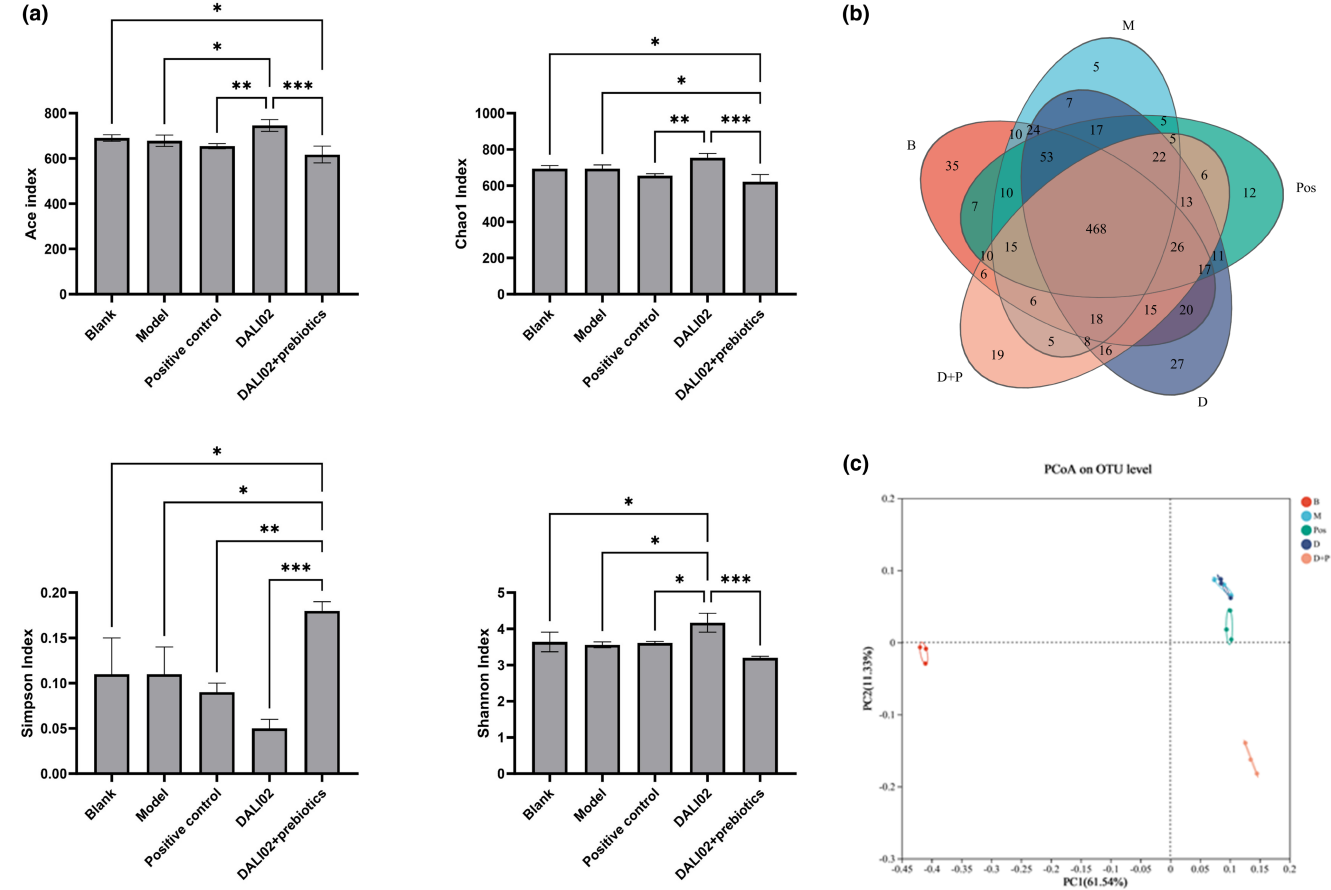
Diversity of gut microbiota. (a) Ace index, Chao 1 index, Simpson index, Shannon index. (b) Principal coordinate analysis of intestinal‐derived microbiota. (c) The OTU distribution Venn diagram. All data are means ± SEM, *n* = 5 per group. *, **, *** Between different groups represents a significant difference (*p* < .05).

After 4 weeks of intervention, the contents of rat colon were taken to detect the intestinal flora. Based on the sequencing of 16S ribosomal RNA (rRNA) amplifiers, the structure of intestinal microbiota in different groups was described by α‐diversity index and principal coordinate analysis (PCoA). The results are shown in Figure 4a, including Ace index, Chao 1 index, Simpson index, and Shannon index at the operational taxonomic unit (OTU) level. There was no significant difference between the Ace index, Chao1 index, Simpson index, and Shannon index of the model group and the blank group (*p* > .05), indicating that cyclophosphamide had little effect on the α‐diversity of rat intestinal flora. Compared with the model group, the Ace and Shannon indices in the DALI02 group were significantly increased (*p* < .05), and the Simpson index was significantly decreased (*p* < .05). In contrast, Chao1 index significantly decreased (*p* < .05) and Simpson index significantly increased (*p* < .05) in the DALI02 + Prebiotics group. This suggested that DALI02 significantly increased the α‐diversity of rat intestinal flora when acting alone (*p* < .05).

As shown in Figure 4b, there were 463 shared OTUs among all groups of rats. The number of unique OTUs for the DALI02 + Prebiotics and DALI02 groups was 19 and 22, respectively, both of which were higher than that of the model group but lower than that of the blank group. The intervention of DALI02 + Prebiotics and DALI02 increased the number of OTUs unique to the rat intestinal flora. The difference of OUTs in rat intestinal flora was shown by principal coordinate analysis (PCoA) based on unweighted UniFrac distance (Figure 4c). The figure showed that the model group, positive control group, and DALI02 group were close to each other and belonged to the first quadrant. The DALI02 + Prebiotics group was in the fourth quadrant, far away from the colonies of the model group, indicating that it had a strong regulatory effect on the changes in intestinal flora diversity caused by cyclophosphamide.

### 3.4 Dynamic changes in gut microbiota composition

**FIGURE 5:**
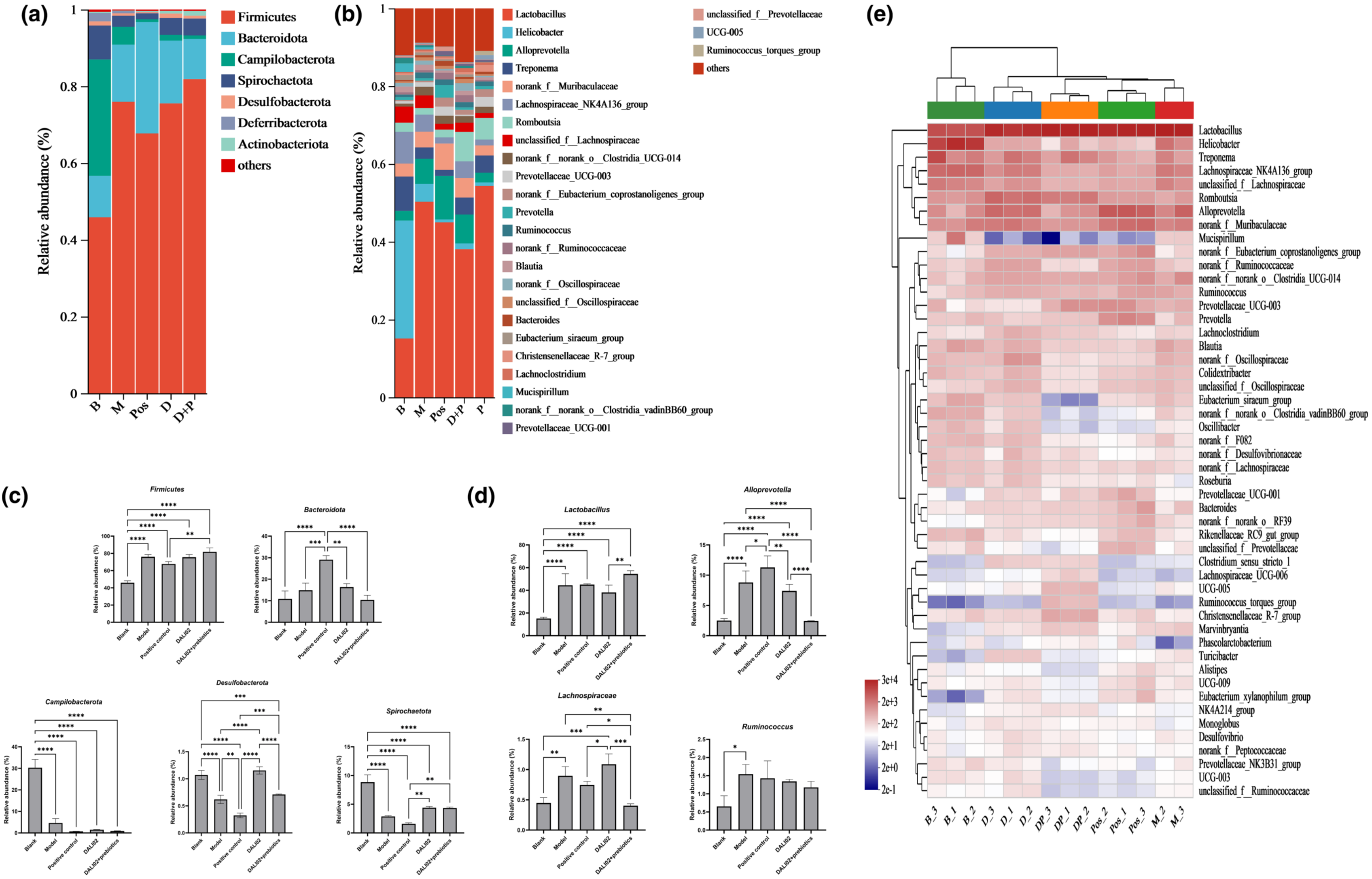
Changes in the structure of intestinal flora in immunocompromised rats. (a) Differences in intestinal flora at the phylum level. (b) Differences in intestinal flora at the genus level. (c) Differences in intestinal flora at the phylum level. (d) Differences in intestinal flora at the genus level. (e) Heatmap and cluster analysis on genus level. All data are means ± SEM, *n* = 5 per group. *, **, ***, **** Between different groups represents a significant difference (*p* < .05).

After the 4‐week intervention, many changes occurred in the composition of the microbial community structure, as shown in Figure 5. The gut microbiota of rats at the phylum level was primarily composed of *Firmicutes*, *Bacteroidota*, *Campilobacterota*, *Spirochaetota*, *Desulfobacterota*, *Actinobacteriota*, *Deferribacterota*, and a few unnamed phyla. Among them, *Firmicutes* and *Campilobacterota* had the highest proportions, at around 45% and 40%, respectively, and were considered the dominant phyla. Following these were *Bacteroidota*, *Spirochaetota*, and *Desulfobacterota*. The remaining phyla constituted a smaller proportion, typically below 1% (Figure 5a). As illustrated in Figure 5c, the relative abundance of *Firmicutes* in the rat gut significantly increased in the model group (*p* < .05), while the relative abundance of *Campilobacterota*, *Desulfobacterota*, and *Spirochaetota* significantly decreased (*p* < .05). Following interventions with DALI02 and DALI02 + Prebiotics, the relative abundance of *Firmicutes* in the rat gut did not change significantly, but it remained significantly higher than that in the blank group (*p* < .05). Furthermore, there was no significant difference in the relative abundance of *Bacteroidota* between the two intervention groups and the blank group (*p* > .05). The relative abundance of *Spirochaetota* increased compared to the model group, but this increase was not statistically significant (*p* > .05). Compared to DALI02 + Prebiotics, DALI02 alone can significantly increase the relative abundance of *Desulfobacterota* (*p* < .05).

As shown in Figure 5b, many changes occurred at the genus level in the gut microbiota. These included an increase in the relative abundance of *Lactobacillus* and *Alloprevotella* (*p* < .05), and a decrease in the relative abundance of *Helicobacter*, *Treponema*, *Romboutsia*, and so on. After the intervention, many changes occurred within the microbial community as well (Figure 5d). Specifically, following interventions with DALI02 and DALI02 + Prebiotics, the level of the *Lactobacillus* remained at a significantly higher level compared to the blank group (*p* < .05). And the relative abundance of *Alloprevotella* significantly decreased (*p* < .05). In the DALI02 + Prebiotics group, the level of *Alloprevotella* was reduced to a level comparable to that of the blank group. Furthermore, following CTX injection, the content of *Lachnospiraceae* and *Ruminococcus* in the model group significantly increased (*p* < .05). There was no significant difference after DALI02 intervention (*p >* .05). However, the relative abundance of *Lachnospiraceae* in the DALI02 + Prebiotics group significantly decreased (*p* < .05).

To holistically investigate the impact of DALI02 and DALI02 + Prebiotics on the gut microbiota structure at the genus level, a cluster analysis was conducted on the gut microbiota of different groups at the genus level. As shown in Figure 5e, the model group was most closely related to the positive control group, while DALI02 and DALI02 + Prebiotics were more similar to the blank group. This suggested that the intervention of DALI02 and DALI02 + Prebiotics can effectively regulate the imbalance of gut microbiota structure caused by immunosuppression.

### 3.5 Modifications of SCFAs content

**FIGURE 6:**
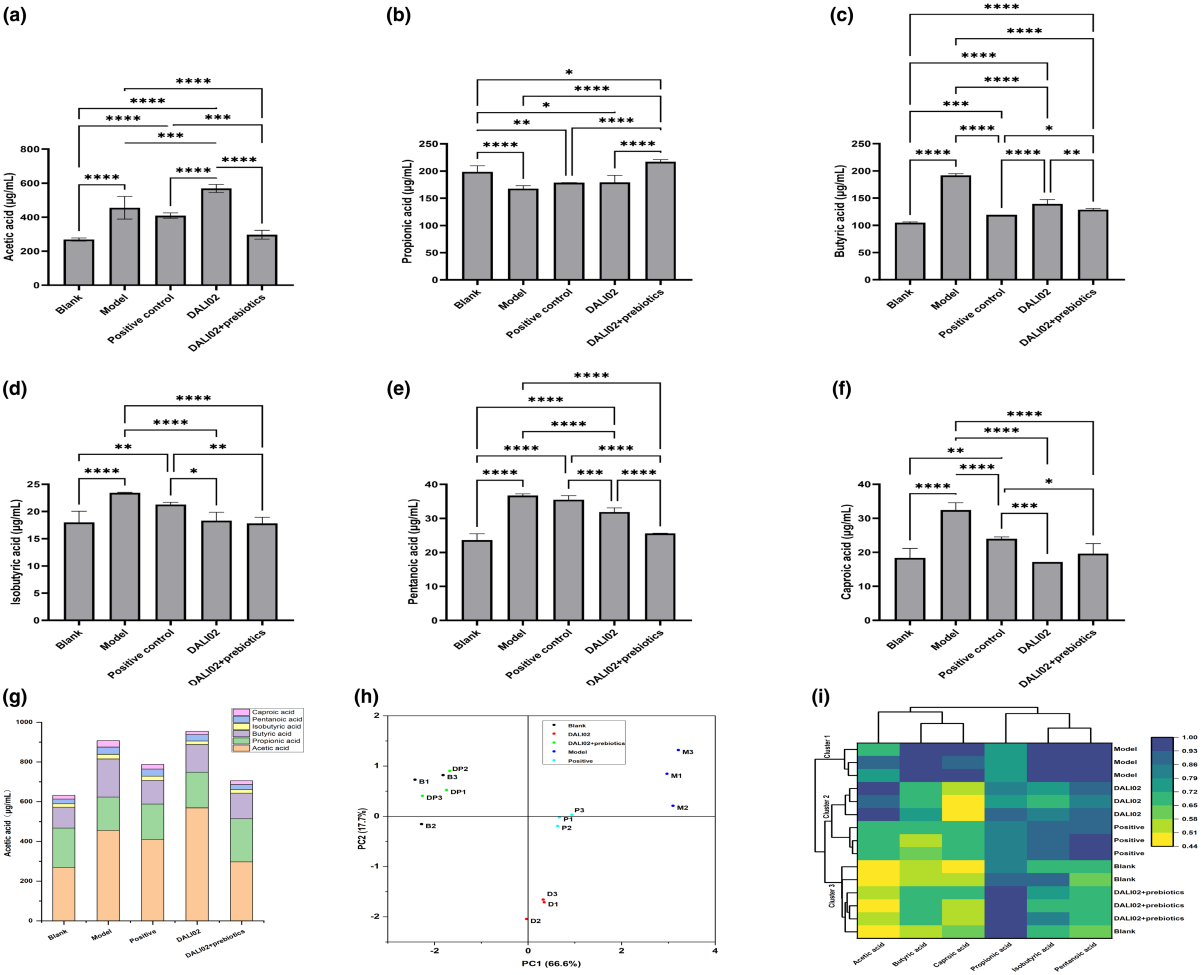
Changes in the short‐chain fatty acid (SCFA) content. (a) Acetic acid. (b) Propionic acid. (c) Butyric acid. (d) Isobutyric acid. (e) Pentanoic acid. (f) Caproic acid. (g) Total content. (h) PCA. (i) Heatmap and cluster analysis. All data are means ± SEM, *n* = 5 per group. *, **, ***, **** Between different groups represents a significant difference (*p* < .05).

After a 4‐week intervention, fresh rat feces were collected, and the content of SCFAs was determined, with the results shown in Figure 6. Compared to the blank group, the total amount of SCFAs in the model group was increased, with significant increases in the levels of acetic acid, butyric acid, isobutyric acid, pentanoic acid, and caproic acid (*p* < .05), while the level of propionic acid significantly decreased (*p* < .05). Intervention with DALI02 and DALI02 + Prebiotics can significantly reduce the content of butyric acid, isobutyric acid, pentanoic acid, and caproic acid (*p* < .05). Intervention with DALI02 + Prebiotics reduced the total content of SCFAs, and DALI02 + Prebiotics intervention significantly increased the content of propionic acid in immunosuppressed rats compared to the intervention with the single DALI02 strain (*p* < .05).

Principal component analysis (PCA) and heatmap and cluster analysis were conducted on the data of SCFAs, with the results shown in Figure 6h,i. First principal component (PC1) and second principal component (PC2) account for 84.3%, indicating that the first and second principal components can effectively represent the conditions of the various SCFAs. The blank group and the DALI02 + Prebiotics group were primarily distributed in the second quadrant, while the model group was mainly distributed in the first quadrant. Cluster analysis revealed that the model group was clustered in Cluster 1, while the blank group and the DALI02 + Prebiotics group were clustered in Cluster 3. The remaining groups were clustered in Cluster 2, and compared to Cluster 1, Cluster 2 showed a higher degree of phylogenetic similarity to Cluster 3. This indicated that the interventions with the single strain DALI02, DALI02 + Prebiotics, and the positive control drug can effectively regulate the dysregulation of SCFAs caused by cyclophosphamide. Furthermore, DALI02 + Prebiotics exhibited a better regulatory effect than the single strain alone.

## 4 DISCUSSION

Regulating immune function to reduce systemic inflammatory responses is one of the main functions of probiotics. Probiotics, via colonizing the gut, alter the intestinal environment and microbial community structure, thereby achieving their diverse beneficial functions. Previous study has optimized the combination of prebiotics that can enhance the adhesion capabilities of *L. fermentum* DALI02 both in vitro and in vivo (Liu et al., 2023). The compound prebiotics can improve the colonization ability of DALI02 in vivo and prolong its residence time in the intestinal tract. This may promote the probiotic function of DALI02.

Based on the previous research results, this study further determined the immunomodulatory effect of DALI02 and explored the effect of prebiotics on the immunomodulatory effect of DALI02. The results indicated that both DALI02 and DALI02 + Prebiotics can effectively alleviate the immunosuppressed state in rats. Furthermore, the intervention with DALI02 + Prebiotics showed a more comprehensive and significant effect. In addition, both DALI02 and DALI02 + Prebiotics can optimize the intestinal microecological environment, including intestinal barrier function, gut flora, and the content and structure of SCFAs. Moreover, the indexes of the DALI02 + Prebiotics group were closer to those of the blank group after intervention.

CTX is an immunosuppressive agent, and its administration can cause damage or even death to immune cells, as well as suppress cell‐mediated immunity and humoral immunity. This study observed similar results, where the administration of CTX suppressed the weight gain in rats (Figure 2a), disrupted the immune function in rats, including a decrease in the thymus index (Figure 2b), inhibition of lymphocyte proliferation (Figure 2c), and stimulation of the systemic humoral immune state (Figure 2e,f). The intervention with DALI02 significantly enhanced lymphocyte proliferation, the A/G ratio, and IL‐6 levels, and significantly reduced TNF‐α levels (*p* < .05). This is consistent with previous research, where dietary supplementation with probiotics can activate the immune system and reduce inflammatory responses. Garcia‐Castillo et al. found that *Lactobacillus* fermentations can activate host immune response by activating the proliferation and differentiation of intestinal macrophages and increasing the secretion of IL‐6 and IgA, which is helpful to the normalization of immune system (Garcia‐Castillo et al., 2019). In addition, DALI02 + Prebiotics can also restore body weight, increase thymus index, increase IgM content, and so on. Thus, it is understandable that DALI02 + Prebiotics has a more comprehensive and superior effect compared to single DALI02.

In addition to severe immunosuppression, cyclophosphamide also alters the population of opportunistic pathogens in the intestine, disrupts intestinal barrier function, and increases intestinal permeability (Yang et al., 2013). In this study, the same results were observed, the cavity area, villi, recess, and goblet cells in the model group changed, and inflammatory cell infiltration appeared in the intestinal tract. In addition, the contents of LPS and D‐lactic acid increased significantly, indicating that the intestinal barrier function was impaired and harmful substances in the intestine entered the blood, which provided conditions for the stimulation of systemic inflammation. Previous studies have shown that both probiotics and probiotics can restore barrier function (Ma et al., 2023; Rose et al., 2021). In this study, the contents of LPS and D‐lactic acid in the intervention group decreased significantly, and the regulatory effect of DALI02 + Prebiotics was particularly significant (*p* < .05).

The gut microbiota and its metabolites, SCFAs, have an important connection with the host immune status (Shin et al., 2023; Wang et al., 2021). Therefore, after discovering the significant immunomodulatory effects of DALI02 and DALI02 + Prebiotics in this study (*p* < .05), the study specifically explored the impact of both on the gut microbiota and SCFAs in immunosuppressed rats. We found that the Chao1 index, ACE, Simpson index, and Shannon index in the model group showed no significant difference compared to the blank group (*p* > .05). After the intervention, the α‐diversity in the DALI02 group significantly increased (*p* < .05), while the α‐diversity in the DALI02 + Prebiotics group significantly decreased (*p* < .05). Consistent with this study, Israr Khan et al. also found that the α‐diversity indices in the immunosuppressed group did not exhibit a significant decrease (Khan et al., 2022), but the intervention with *L. fermentum* was able to enhance the Chao 1 index and Shannon diversity index of the gut microbiota (Rodriguez‐Nogales et al., 2017). However, contrary to other research findings, this study found that the intake of synbiotics significantly reduced α‐diversity (Zhao et al., 2021). The results of the PCoA indicated that while the DALI02 + Prebiotics group did not cluster closely with the blank group, it was distinct from the model group.

More specifically, we observed significant changes in the microbial community at the phylum and genus levels, particularly in the *Firmicutes* phylum (*p* < .05). The majority of the bacteria within the *Firmicutes* phylum are beneficial, such as *Faecalibacterium* and *Lactobacillus*. They play a significant role in intestinal health. Considering that this composite prebiotics can significantly promote the adhesion and proliferation of *L. fermentum* DALI02 (Liu et al., 2023), it is predictable that the relative abundance of the *Lactobacillus* genus and the *Firmicutes* phylum in the gut in the DALI02 + Prebiotics group would be significantly increased (*p* < .05) (Figure 5). *Alloprevotella* possesses key enzymes for the degradation of mucin proteins (Wang, Meng, et al., 2024); hence, the restoration of intestinal permeability in the intervention group may be associated with its reduced abundance. The abundance of *Ruminococcus* is positively correlated with intestinal inflammation and colorectal cancer (Wan et al., 2023). In this study, the content of *Ruminococcus* in the model group was significantly higher than that in the blank group (*p* < .05), and a reduction was observed in the content across all intervention groups. However, the increase in the *Firmicutes* phylum and the *Lactobacillus* genus in the model group was unexpected. This may be due to the gut microbiota being stimulated by CTX, and after 4 weeks, the gut microbiota began to self‐recover. This finding is corroborated by previous research, where Israr Khan discovered that the *Firmicutes* content in the model group significantly increased at 30 days, but this trend was not observed within the first 21 days postmodeling (Khan et al., 2022).

As an energy source for colonocytes, numerous studies have indicated that SCFAs can maintain intestinal health, repair damaged intestinal mucosa, and modulate the microbial community structure (You et al., 2022). In this study, the intervention with DALI02 was able to increase the overall content of SCFAs, particularly significantly increasing the content of acetic acid (*p* < .05), while DALI02 + Prebiotics significantly increased the content of propionic acid (*p* < .05). This is consistent with previous research findings, where the probiotic *Bacillus subtilis* significantly increased the total content of SCFAs and the level of acetic acid in piglets (Yu et al., 2022). And the intake of the prebiotic inulin can increase the level of propionic acid in mouse feces. However, distinctively, this study observed a significant increase in the levels of total SCFAs, acetic acid, butyric acid, isobutyric acid, pentanoic acid, and caproic acid in the model group (*p* < .05). This is consistent with the results of the gut microbiota analysis. This may be due to the self‐repair in response to cyclophosphamide over a period of 4 weeks, but this repair was limited to the intestinal environment. The immune status and intestinal barrier function of the model group remain in a poor state (Figures 2, 3, 4, 5). Through the analysis of the level of intestinal flora and SCFAs by PCA and cluster analysis, we observed that there was a high affinity among DALI02 + Prebiotics, DALI02 group, and blank group. For SCFAs, DALI02 + Prebiotics and the blank group were clustered together (Figure 6h) and belonged to Cluster 3 after clustering (Figure 6i). These findings indicated that interventions with DALI02 and DALI02 + Prebiotics may effectively counteract the dysbiosis of gut microbiota structure and SCFAs induced by immunodeficiency, with the intervention of DALI02 + Prebiotics showing greater efficacy.

## 5 CONCLUSION

In summary, *L. fermentum* DALI02 and the combination of DALI02 with fermented composite prebiotics exhibited excellent immunomodulatory functions, with DALI02 + Prebiotics showing a more comprehensive and pronounced regulatory effect, including the restoration of body weight, enhancement of the thymus index, improvement of splenocyte proliferation, and reduction of IL‐6 and TNF‐α secretion. Furthermore, DALI02 and DALI02 + Prebiotics were capable of restoring intestinal barrier function, modulating the microbial community structure, and regulating the levels and composition of SCFAs. These results substantiated the immunomodulatory effects of DALI02 as a probiotic and provided a composite prebiotic combination that enhanced the functionality of the strain, offering new insights into the development of immunomodulatory synbiotic functional foods.

## AUTHOR CONTRIBUTIONS

**Longfei Zhang:** Conceptualization (lead); formal analysis (lead); funding acquisition (lead); methodology (lead); writing – original draft (lead); writing – review and editing (lead). **Xiaoxiao Liu:** Data curation (supporting); investigation (supporting); software (lead); supervision (supporting); writing – original draft (supporting); writing – review and editing (supporting). **Yang Liu:** Data curation (supporting); methodology (supporting); project administration (supporting); validation (supporting). **Xinyi Cheng:** Data curation (supporting); project administration (supporting); validation (supporting). **Mingze Xu:** Conceptualization (supporting); funding acquisition (lead); resources (lead). **Hengxian Qu:** Formal analysis (supporting); funding acquisition (supporting); supervision (supporting); validation (supporting). **Wenqiong Wang:** Conceptualization (supporting); resources (lead); software (supporting). **Ruixia Gu:** Conceptualization (supporting); formal analysis (supporting); funding acquisition (supporting); resources (lead); visualization (supporting). **Dawei Chen:** Conceptualization (supporting); funding acquisition (supporting); project administration (supporting); resources (lead); supervision (lead); validation (supporting); visualization (supporting).

## FUNDING INFORMATION

This research was funded by the National Key Research and Development Program of China for the 14th Five‐year Plan, grant number 2022YFD2101503; the Project of National Natural Science Foundation of China, grant numbers 31972094 and 32272362; The Natural Science Foundation of Jiangsu Province, grant number BK20211325; City‐school cooperation to build a scientific and technological innovation platform project, grant number YZ2020265; Postgraduate Research & Practice Innovation Program of Jiangsu Province, grant number KYCX22_3500.

## CONFLICT OF INTEREST STATEMENT

No conflict of interest exists in the submission of this manuscript, and the manuscript is approved by all authors for publication.

## Data Availability

Research data are not shared.

## References

[fsn34361-bib-0001] Feng, Y. , Wang, Y. , Wang, P. , Huang, Y. , & Wang, F. (2018). Short‐chain fatty acids manifest stimulative and protective effects on intestinal barrier function through the inhibition of NLRP3 inflammasome and autophagy. Cellular Physiology and Biochemistry, 49, 190–205. 30138914 10.1159/000492853

[fsn34361-bib-0002] Garcia‐Castillo, V. , Komatsu, R. , Clua, P. , Indo, Y. , Takagi, M. , Salva, S. , Islam, M. A. , Alvarez, S. , Takahashi, H. , Garcia‐Cancino, A. , Kitazawa, H. , & Villena, J. (2019). Evaluation of the immunomodulatory activities of the probiotic strain *Lactobacillus fermentum* UCO‐979C. Frontiers in Immunology, 10, 1376. 31263467 10.3389/fimmu.2019.01376 PMC6585165

[fsn34361-bib-0003] Hill, C. , Guarner, F. , Reid, G. , Gibson, G. R. , Merenstein, D. J. , Pot, B. , Morelli, L. , Canani, R. B. , Flint, H. J. , Salminen, S. , Calder, P. C. , & Sanders, M. E. (2014). The international scientific association for probiotics and prebiotics consensus statement on the scope and appropriate use of the term probiotic. Nature Reviews Gastroenterology & Hepatology, 11, 506–514. 24912386 10.1038/nrgastro.2014.66

[fsn34361-bib-0004] Jorjao, A. L. , De Oliveira, F. E. , Leao, M. V. P. , Carvalho, C. A. T. , Jorge, A. O. C. , & De Oliveira, L. D. (2015). Live and heat‐killed *Lactobacillus rhamnosus* ATCC 7469 may induce modulatory cytokines profiles on macrophages RAW 264.7. The Scientific World Journal, 2015, 716749. 26649329 10.1155/2015/716749 PMC4663741

[fsn34361-bib-0005] Khan, I. , Wei, J. , Li, A. , Liu, Z. , Yang, P. , Jing, Y. , Chen, X. , Zhao, T. , Bai, Y. , Zha, L. , Li, C. , Ullah, N. , Che, T. , & Zhang, C. (2022). *Lactobacillus plantarum* strains attenuated DSS‐induced colitis in mice by modulating the gut microbiota and immune response. International Microbiology, 25, 587–603. 35414032 10.1007/s10123-022-00243-y

[fsn34361-bib-0006] Kim, J.‐K. , Kim, J.‐Y. , Kim, H. I. , Han, M. J. , & Kim, D.‐H. (2019). *Bifidobacterium longum* and *Lactobacillus plantarum* alleviate house dust mite allergen‐induced allergic rhinitis by regulating IL‐4, IL‐5, and IL‐10 expression. Food and Agricultural Immunology, 30, 581–593.

[fsn34361-bib-0007] Liu, X. , Chen, D. , Li, Q. , Zhang, C. , Zhang, L. , Qu, H. , Wang, W. , Zhou, Y. , Huang, Y. , Xiao, L. , & Gu, R. (2023). Effect of complex prebiotics on the intestinal colonization ability of *Limosilactobacillus fermentum* DALI02. Fermentation‐Basel, 9, 25.

[fsn34361-bib-0008] Ma, W. , Li, W. , Yu, S. , Bian, H. , Wang, Y. , Jin, Y. , Zhang, Z. , Ma, Q. , & Huang, L. (2023). Immunomodulatory effects of complex probiotics on the immuno‐suppressed mice induced by cyclophosphamide. Frontiers in Microbiology, 14, 1055197. 36778877 10.3389/fmicb.2023.1055197 PMC9911820

[fsn34361-bib-0009] Rodriguez‐Nogales, A. , Algieri, F. , Garrido‐Mesa, J. , Vezza, T. , Utrilla, M. P. , Chueca, N. , Garcia, F. , Olivares, M. , Rodríguez‐Cabezas, M. E. , & Gálvez, J. (2017). Differential intestinal anti‐inflammatory effects of *Lactobacillus fermentum* and *Lactobacillus salivarius* in DSS mouse colitis: Impact on microRNAs expression and microbiota composition. Molecular Nutrition & Food Research, 61, 1700144. 10.1002/mnfr.201700144 28752563

[fsn34361-bib-0010] Rose, E. C. , Odle, J. , Blikslager, A. T. , & Ziegler, A. L. (2021). Probiotics, prebiotics and epithelial tight junctions: A promising approach to modulate intestinal barrier function. International Journal of Molecular Sciences, 22, 6729. 34201613 10.3390/ijms22136729 PMC8268081

[fsn34361-bib-0011] Shin, Y. , Han, S. , Kwon, J. , Ju, S. , Choi, T. , Kang, I. , & Kim, S. (2023). Roles of short‐chain fatty acids in inflammatory bowel disease. Nutrients, 15, 4466. 37892541 10.3390/nu15204466 PMC10609902

[fsn34361-bib-0012] Vera‐Santander, V. E. , Hernandez‐Figueroa, R. H. , Jimenez‐Munguia, M. T. , Mani‐Lopez, E. , & Lopez‐Malo, A. (2023). Health benefits of consuming foods with bacterial probiotics, postbiotics, and their metabolites: A review. Molecules, 28, 1230. 36770898 10.3390/molecules28031230 PMC9920731

[fsn34361-bib-0013] Wan, J. , Yu, X. , Liu, J. , Li, J. , Ai, T. , Yin, C. , Liu, H. , & Qin, R. (2023). A special polysaccharide hydrogel coated on *Brasenia schreberi*: Preventive effects against ulcerative colitis via modulation of gut microbiota. Food & Function, 14, 3564–3575. 36946057 10.1039/d2fo03207d

[fsn34361-bib-0014] Wang, D. , Zeng, J. , Wujin, C. , Ullah, Q. , & Su, Z. (2024). *Lactobacillus reuteri* derived from horse alleviates *Escherichia coli*‐induced diarrhea by modulating gut microbiota. Microbial Pathogenesis, 188, 106541. 38224920 10.1016/j.micpath.2024.106541

[fsn34361-bib-0015] Wang, X. , Meng, M. , Sun, J. , Gao, W. , Lin, C. , & Yu, C. (2024). *Klebsiella aerogenes* exacerbates colon tumorigenesis in the AOM/DSS‐induced C57BL/6J mouse. Biochemical and Biophysical Research Communications, 694, 149410. 38134478 10.1016/j.bbrc.2023.149410

[fsn34361-bib-0016] Wang, X. , Zhang, P. , & Zhang, X. (2021). Probiotics regulate gut microbiota: An effective method to improve immunity. Molecules, 26, 6076. 34641619 10.3390/molecules26196076 PMC8512487

[fsn34361-bib-0017] Xu, J. , Wang, R. , Zhang, H. , Wu, J. , Zhu, L. , & Zhan, X. (2021). In vitro assessment of prebiotic properties of oligosaccharides derived from four microbial polysaccharides. LWT – Food Science and Technology, 147, 111544. 10.1080/09637486.2021.1908964 33870850

[fsn34361-bib-0018] Yang, J. , Liu, K.‐X. , Qu, J.‐M. , & Wang, X.‐D. (2013). The changes induced by cyclophosphamide in intestinal barrier and microflora in mice. European Journal of Pharmacology, 714, 120–124. 23791611 10.1016/j.ejphar.2013.06.006

[fsn34361-bib-0019] You, S. , Ma, Y. , Yan, B. , Pei, W. , Wu, Q. , Ding, C. , & Huang, C. (2022). The promotion mechanism of prebiotics for probiotics: A review. Frontiers in Nutrition, 9, 1000517. 36276830 10.3389/fnut.2022.1000517 PMC9581195

[fsn34361-bib-0020] Yu, S. , Wang, H. , Cui, L. , Wang, J. , Zhang, Z. , Wu, Z. , Lin, X. , He, N. , Zou, Y. , & Li, S. (2023). Pectic oligosaccharides ameliorate high‐fat diet‐induced obesity and hepatic steatosis in association with modulating gut microbiota in mice. Food & Function, 14, 9892–9906. 37853813 10.1039/d3fo02168h

[fsn34361-bib-0021] Yu, X. , Cui, Z. , Qin, S. , Zhang, R. , Wu, Y. , Liu, J. , & Yang, C. (2022). Effects of *Bacillus licheniformis* on growth performance, diarrhea incidence, antioxidant capacity, immune function, and fecal microflora in weaned piglets. Animals, 12, 1609. 35804509 10.3390/ani12131609 PMC9264952

[fsn34361-bib-0022] Yue, Y. , He, Z. , Zhou, Y. , Ross, R. P. , Stanton, C. , Zhao, J. , Zhang, H. , Yang, B. , & Chen, W. (2020). *Lactobacillus plantarum* relieves diarrhea caused by enterotoxin‐producing *Escherichia coli* through inflammation modulation and gut microbiota regulation. Food & Function, 11, 10362–10374. 33220669 10.1039/d0fo02670k

[fsn34361-bib-0023] Zeng, Z. , Huang, Z. , Yue, W. , Nawaz, S. , Chen, X. , & Liu, J. (2023). *Lactobacillus plantarum* modulate gut microbiota and intestinal immunity in cyclophosphamide‐treated mice model. Biomedicine & Pharmacotherapy, 169, 115812. 37979376 10.1016/j.biopha.2023.115812

[fsn34361-bib-0024] Zhang, B. , Zhong, Q. , Liu, N. , Song, P. , Zhu, P. , Zhang, C. , & Sun, Z. (2022). Dietary glutamine supplementation alleviated inflammation responses and improved intestinal mucosa barrier of LPS‐challenged broilers. Animals, 12, 1729. 35804628 10.3390/ani12131729 PMC9265045

[fsn34361-bib-0025] Zhao, S. , Peng, X. , Zhou, Q.‐Y. , Huang, Y. Y. , Rao, X. , Tu, J. L. , Xiao, H. Y. , & Liu, D. M. (2021). *Bacillus coagulans* 13002 and fructo‐oligosaccharides improve the immunity of mice with immunosuppression induced by cyclophosphamide through modulating intestinal‐derived and fecal microbiota. Food Research International, 140, 109793. 33648160 10.1016/j.foodres.2020.109793

